# One-step nonhydrolytic sol–gel synthesis of mesoporous TiO_2_ phosphonate hybrid materials

**DOI:** 10.3762/bjnano.10.35

**Published:** 2019-02-05

**Authors:** Yanhui Wang, P Hubert Mutin, Johan G Alauzun

**Affiliations:** 1 Institut Charles Gerhardt Montpellier, UMR 5253, Université de Montpellier, CC 1701, Place Eugène Bataillon, 34095 Montpellier Cedex 5, France

**Keywords:** anatase, mesoporous, nonaqueous sol–gel, phosphonate

## Abstract

Mesoporous TiO_2_–octylphosphonate hybrid materials were prepared in one step by a nonhydrolytic sol–gel method involving the reaction of Ti(OiPr)_4_, acetophenone (2 equiv) and diethyl octylphosphonate (from 0 to 0.2 equiv) at 200 °C for 12 hours, in toluene. The different samples were characterized by ^31^P magic angle spinning nuclear magnetic resonance, Fourier transform infrared spectroscopy, Raman spectroscopy, X-ray diffraction, and nitrogen physisorption. For P/Ti ratios up to 0.1, the hybrid materials can be described as aggregated, roughly spherical, crystalline anatase nanoparticles grafted by octylphosphonate groups via Ti–O–P bonds. The crystallite size decreases with the P/Ti ratio, leading to an increase of the specific surface area and a decrease of the pore size of the hybrid samples. For a P/Ti ratio of 0.2, the volume fraction of organic octyl groups exceeds 50%. The hybrid material becomes nonporous and can be described as amorphous TiO_2_ clusters modified by octylphosphonate units, where the octyl chains form an organic continuous matrix.

## Introduction

The development of porous hybrid organic–inorganic materials has been a major goal for materials scientists for more than 25 years [1–3]. Combining inorganic and organic moieties at the nanoscale allows the design of tailor-made functional materials with enhanced or new properties, adapted to a wide range of advanced applications [4–7]. In Class I hybrid materials, the inorganic and organic parts are linked through weak bonds (e.g., van der Waals or hydrogen bonds), while in Class II hybrid materials, they are linked by stronger ionocovalent or covalent bonds [8].

The majority of Class II hybrid materials utilize the stability of the Si–C bond and are based on organosilsesquioxane (R–SiO_1.5_) or bridged organosilsesquioxane (O_1.5_Si–R–O_1.5_). These hybrids are usually prepared by conventional sol–gel processing, i.e., by hydrolysis and condensation of alkoxysilane precursors, which offers an excellent control over composition and homogeneity, while texture can be tuned using various templating approaches [9].

In the case of metals, as M–C bonds are (in most cases) quite unstable, the organic groups can be linked to the metal oxide network via carboxylate or β-diketonate ligands [5,10–11]. Organophosphorus ligands such as phosphonates appear quite promising as they form strong ionocovalent M–O–P bonds with many metals, as shown by the numerous examples of metal phosphonates reported in the literature [12–14]. However, in the case of monophosphonate groups, metal phosphonates usually form semicrystalline layered materials with no interlayer porosity. A way to avoid the formation of a layered material is to use an excess of metal precursor in order to form a metal oxide–phosphonate hybrid material. There are very few examples of the preparation of such materials by sol–gel methods [15–16], and the texture of these materials has not been reported. Actually, most metal oxide–phosphonate-based porous hybrids are obtained in two steps, by surface modification of a porous metal oxide support [17–19].

Nonhydrolytic (or nonaqueous) sol–gel (NHSG) chemistry has provided simple and powerful routes to synthesize oxides or mixed oxides with different morphologies (e.g., nanoparticles) or textures (e.g., mesoporous materials) [20–24]. Several NHSG routes have also been used to prepare Class II hybrids. For instance the alkyl elimination route was applied to the synthesis of organosilsesquioxanes, organosilsesquioxane–metal oxide hybrids [25–27], silica–titania modified by organosilicon groups [28–29], and metal phosphonates [30]. More recently, hybrid silicophosphate xerogels have been produced by reaction of acetoxysilanes with trimethylsilyl esters of phosphoric or phosphonic acids [31], and porous organosilicate covalent polymers have been synthesized by reaction of silicon acetate with 1,3,5-trihydroxybenzene [32].

The reaction of alkoxides in acetophenone (used as a solvent and an oxygen donor) has already been described for the synthesis of TiO_2_ [33] and BaTiO_3_ [34] nanoparticles, but it has never been used to prepare mesoporous oxides or hybrid materials.

In the present work, we present an original one-step NHSG synthesis of mesoporous TiO_2_–octylphosphonate hybrid materials, using a nonhydrolytic sol–gel method involving the reaction of titanium tetraisopropoxide and diethyl octylphosphonate precursors at 200 °C in the presence of acetophenone as an oxygen donor.

## Results

A series of TiO_2_–octylphosphonate hybrids was synthesized by reaction of Ti(OiPr)_4_ (1 equiv) with different amounts of diethyl octylphosphonate (0.02, 0.05, 0.1 and 0.2 equiv) and acetophenone (2 equiv) at 200 °C. It must be mentioned that, in the absence of acetophenone, diethyl octylphosphonate did not react with titanium tetraisopropoxide under the same conditions. All four materials are referred to as TiP*_x_* where *x* is the P/Ti ratio. For comparison, a TiO_2_ sample was prepared under the exact same conditions but without diethyl octylphosphonate.

Elemental analysis by energy dispersive X-ray spectroscopy (EDX) of these materials showed that in all cases the measured P/Ti ratios were close to the nominal ones, indicating that all the octylphosphonate units were incorporated in the materials (Table 1).

**Table 1:** Elemental analysis, crystallite size and textural data for TiO_2_–octylphosphonate hybrids and TiO_2_.

Sample	P/Ti ratio^a^	Cryst. size^b^ (nm)	S_BET_^c^ (m^2^ g^−1^)	*V*_p_^d^ (cm^3^ g^−1^)	*D*_p_^e^ (nm)

TiO_2_	NA	9	120	0.35	9.7
TiP_0.02_	0.023	16	120	0.29	8.0
TiP_0.05_	0.054	11	160	0.23	4.5
TiP_0.1_	0.096	6	240	0.17	3.1
TiP_0.2_	0.192	NA	<10	<0.01	NA

^a^P/Ti ratio determined by EDX; ^b^crystallite size determined by the Scherrer equation for the (101) reflection; ^c^specific surface area, Brunauer–Emmett–Teller (BET) method; ^d^total pore volume at *P*/*P*_0_ = 0.990; ^e^Barrett–Joyner–Halenda (BJH) average pore diameter calculated from the desorption branch. NA: not applicable. ^31^P solid-state nuclear magnetic resonance (NMR) spectroscopy is a useful tool for studying phosphonate-based hybrid materials: it gives information on the presence of phosphonate units bonded to the oxide nework, on the presence of a metal phosphonate phase or of "free" phosphonate precursor (e.g., excess precursor, physisorbed or trapped molecules), but it is not possible to ascribe the different components found in the ^31^P NMR spectrum to the different bonding modes (mono-, bi-, and tridentate phosphonate units) [35].

**Figure 1:**
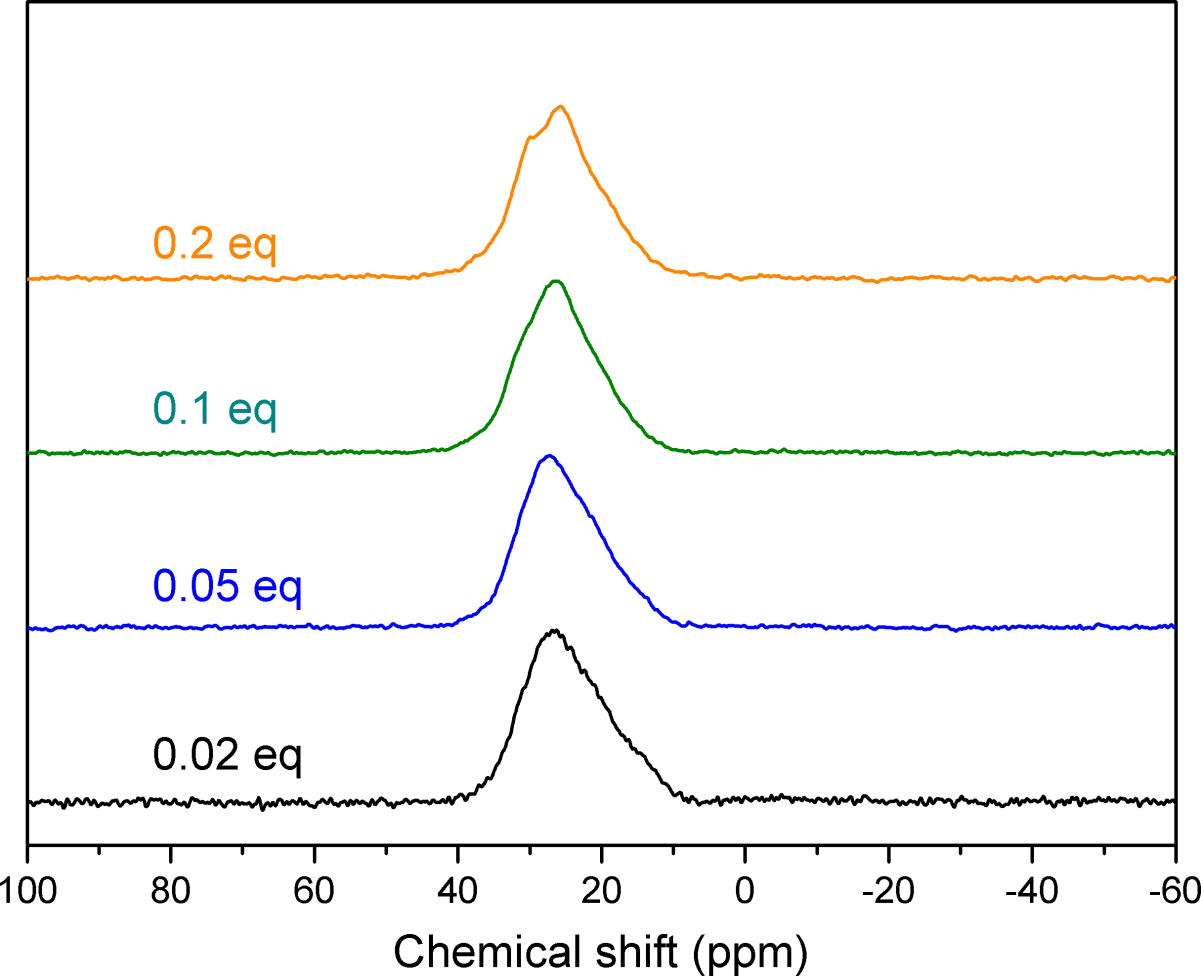
^31^P CP magic angle spinning solid-state NMR spectra of the hybrid materials produced by the reaction of octylphosphonate, Ti(OiPr)_4_ and acetophenone (TiO_2_-octylphosphonate hybrids).

The ^31^P solid-state NMR spectra of the hybrid materials (Figure 1) display a very broad signal in the 10 to 35 ppm range. Similar broad resonances have been reported for TiO_2_–phenylphosphonate hybrid materials prepared in a two-step sol–gel process from Ti(OiPr)_4_ and PhPO_3_H_2_ [15], whereas the hybrid materials obtained by surface modification of anatase supports usually show narrower resonances [36]. These spectra confirm the presence of phosphonate species linked to the TiO_2_ network through Ti–O–P bonds, and show the absence of a layered titanium octylphosphonate phase, which would lead to a sharp resonance at 7 ppm. In the case of the TiP_0.2_ sample, the shoulder at 31 ppm suggests the presence of a small amount of noncondensed diethyl octylphosphonate precursor, possibly molecules trapped in the network of this nonporous sample (see below).

**Figure 2:**
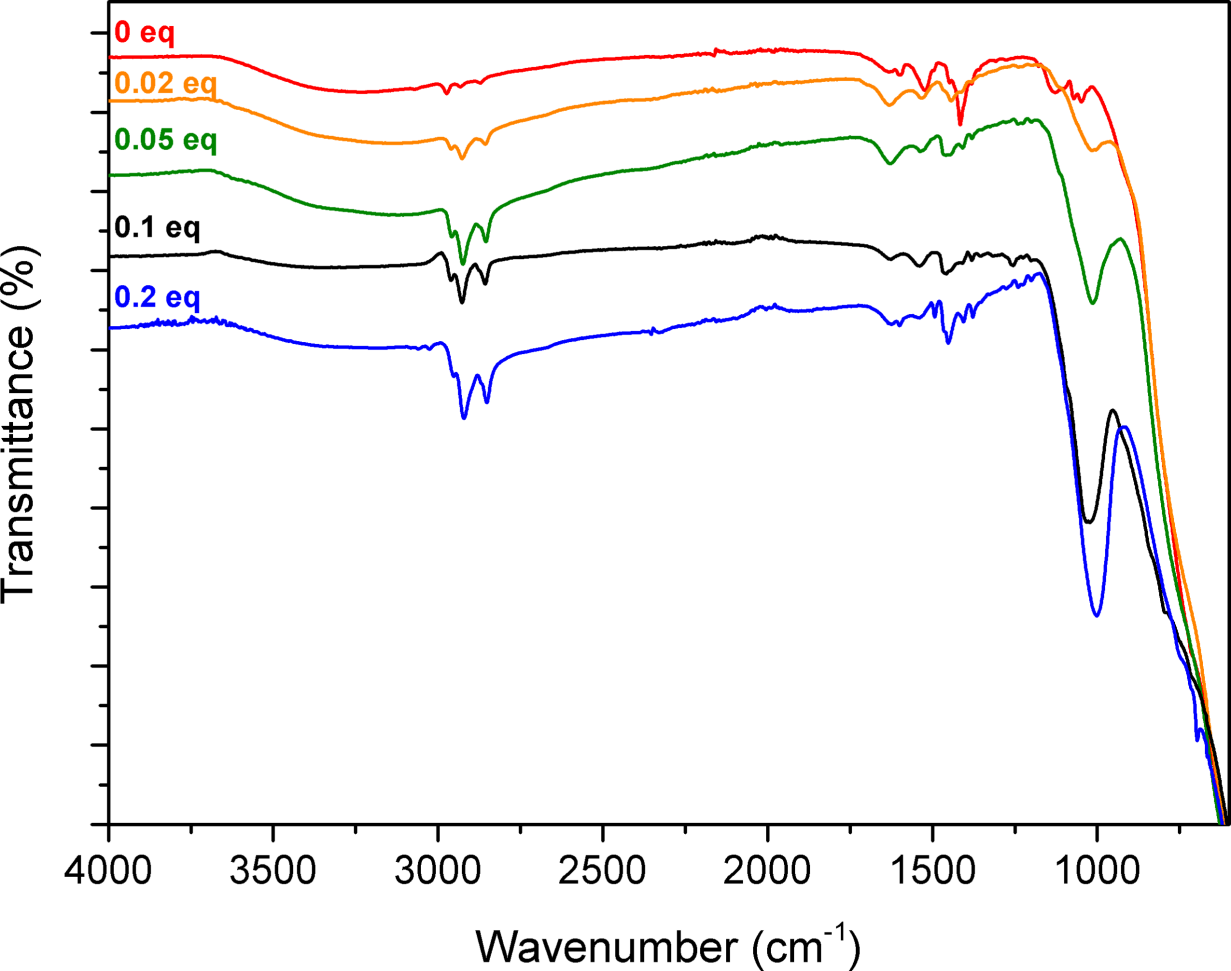
ATR-FTIR spectra of the TiO_2_–octylphosphonate hybrid materials.

The attenuated total reflection (ATR)-Fourier transform infrared (FTIR) spectra of the TiO_2_–octylphosphonate hybrid materials (Figure 2) showed a single broad vibration between 900 and 1200 cm^−1^ arising from vibration modes of the CPO_3_ tetrahedra. The intensity of this band increases with the P/Ti ratio. The absence of bands at ≈1220 cm^−1^ (P=O stretching vibration) and ≈950 cm^−1^ (P–OC stretching vibrations) [36] suggests that most of the phosphonate groups are in the same tridentate environment as in layered titanium phosphonates, that is, bonded to three Ti atoms in CP(OTi)_3_ sites, as previously reported for sol–gel TiO_2_–phenylphosphonate hybrid materials [15]. The vibrations in the 1400–1500 cm^−1^ range can be ascribed to CH_3_ and CH_2_ deformations of groups in residual surface moieties (e.g., Ti–O^i^Pr, Ti–O–CMePhOiPr), and to CH_3_, CH_2_ and P–CH_2_ deformations in the octylphosphonate groups. The three bands between 2800 and 3000 cm^−1^ are ascribed to the C–H symmetric and asymmetric stretching vibrations of bonds in CH_2_ and CH_3_, mostly in the octyl groups, as shown by the intensity of these bands which is directly related to the P/Ti ratio. The weak, broad band between 3000 and 3800 cm^−1^ is characteristic of O–H stretching vibrations. This band indicates the presence of a low amount of adsorbed water (confirmed by the vibration at 1620 cm^−1^ assigned to a deformation mode of adsorbed water), and also of surface hydroxyl groups resulting from the hydrolysis of residual surface groups during washing or manipulation under air.

**Figure 3:**
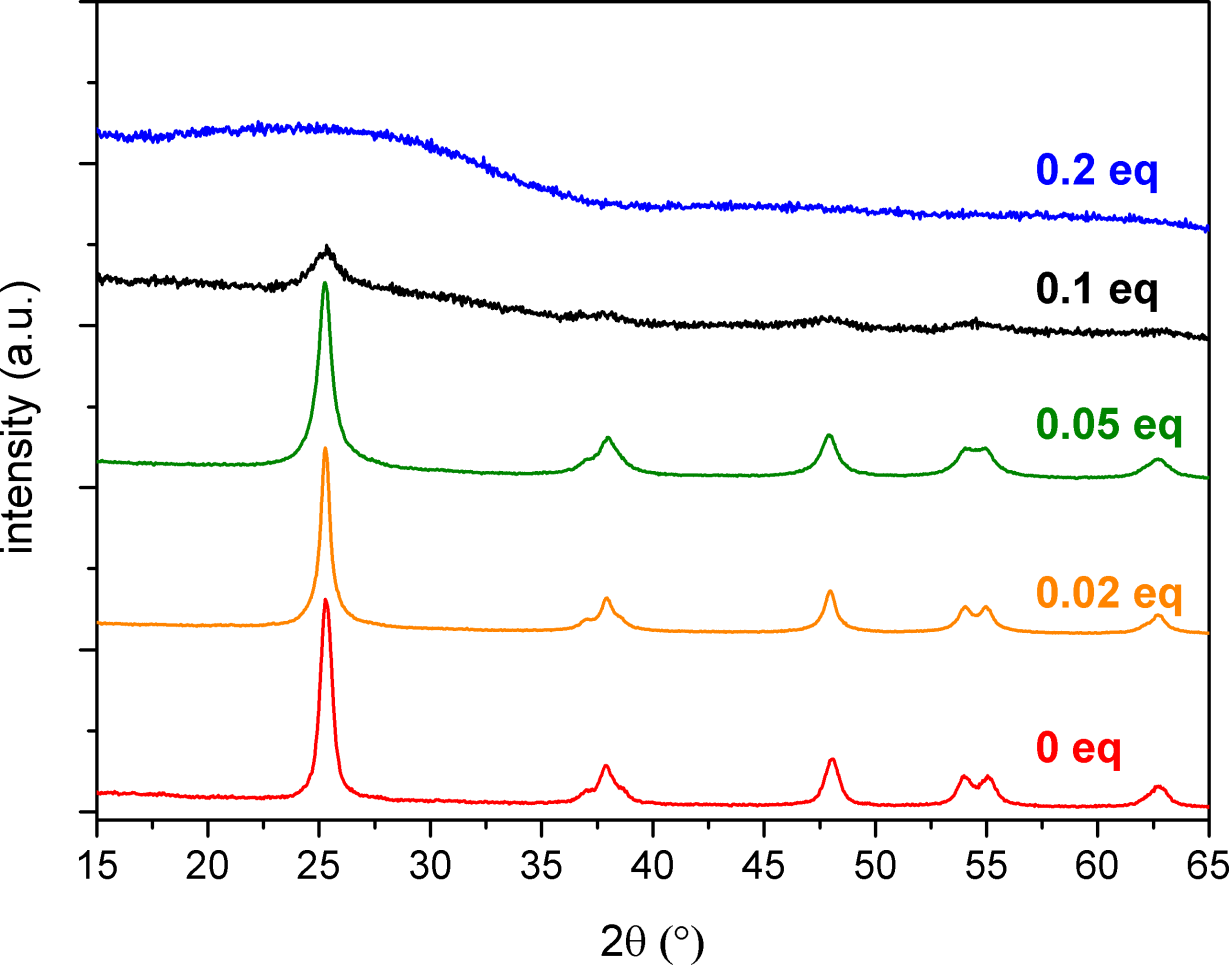
Powder XRD patterns of the TiO_2_–octylphosphonate hybrid materials.

The X-ray diffraction (XRD) patterns of TiO_2_ and of the hybrid samples are presented in Figure 3. The patterns of TiP_0.02_ and TiP_0.05_ showed the presence of well-crystallized anatase nanocrystals (JCPDS 21-1272), as in the TiO_2_ sample. There was no evidence of rutile. The TiP_0.1_ sample appeared partially crystallized, while the TiP_0.2_ sample was amorphous in XRD experiments. The crystallite size (Table 1) of the hybrid samples decreased with the P/Ti ratio from 16 to 6 nm. The lower intensity of the (004) reflection compared to the (200) reflection indicated that the crystallites are not elongated and have a roughly spherical morphology.

**Figure 4:**
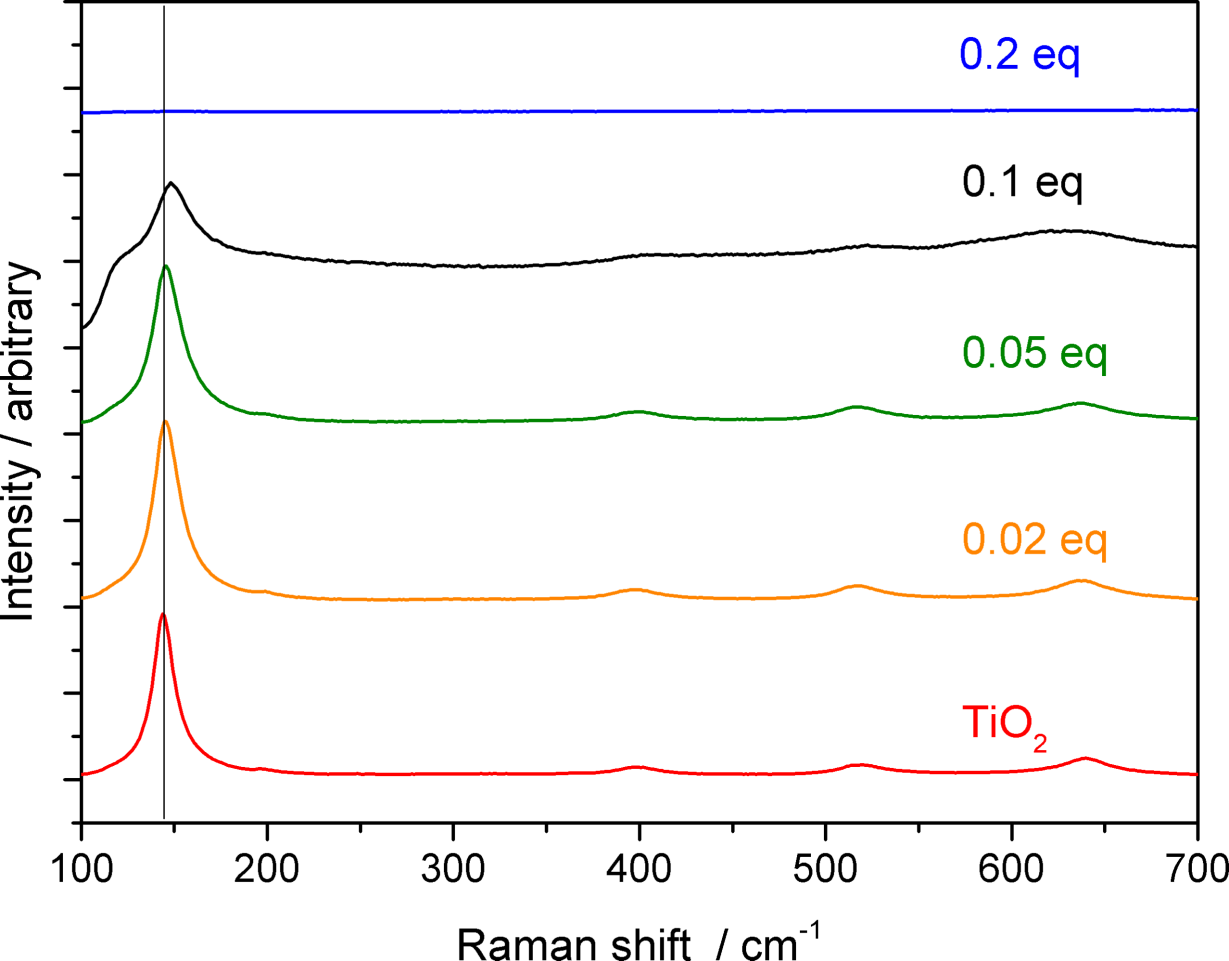
Raman spectra of TiO_2_–octylphosphonate hybrid materials and TiO_2_.

Raman spectroscopy (Figure 4) confirmed the XRD results. For P/Ti ratios between 0 and 0.05, the spectra showed a strong peak at 145 cm^−1^ and smaller peaks at 195, 400, 515 and 640 cm^−1^ indicating the presence of anatase. The spectrum of TiP_0.1_ showed broader and weaker bands, and the first band was shifted to 148 cm^−1^. Under the same conditions, no bands could be observed in the spectrum of TiP_0.2_, indicating highly disordered TiO_2_ domains.

**Figure 5:**
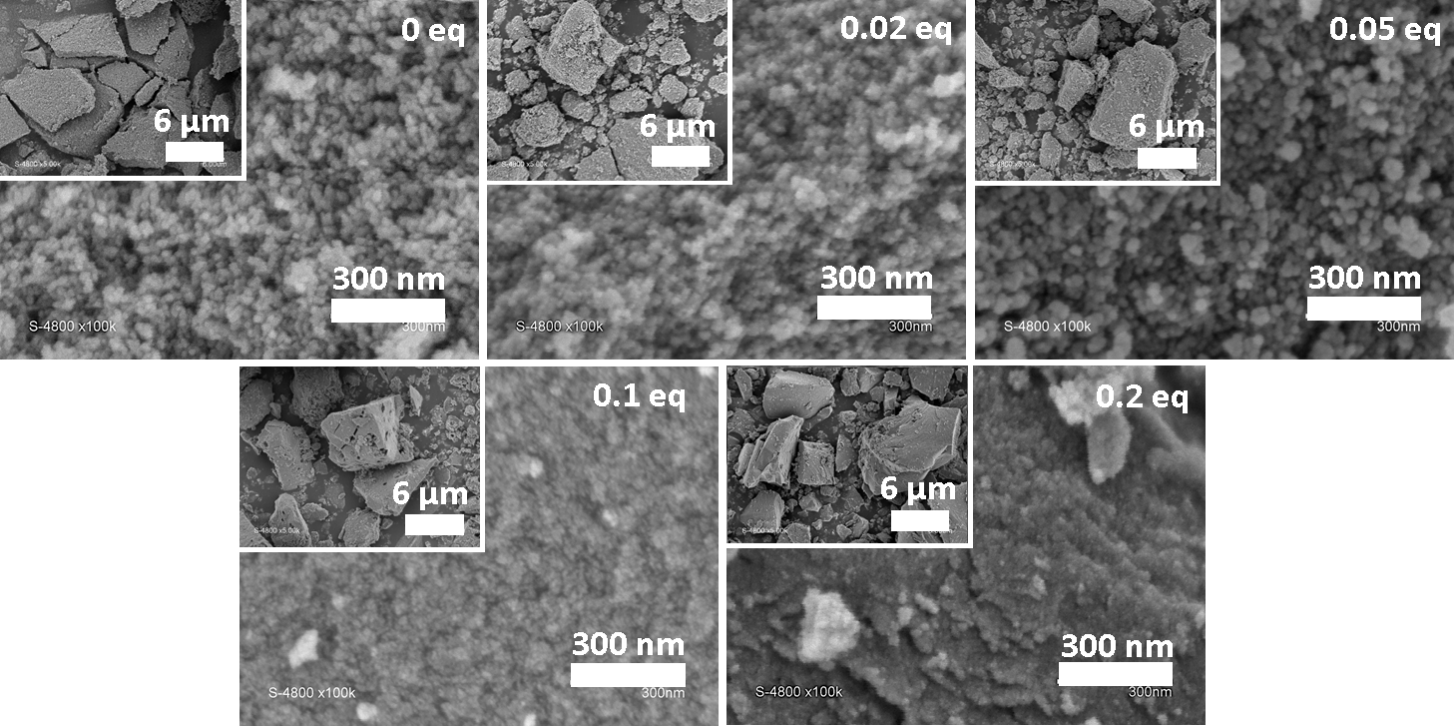
SEM images of TiO_2_–octylphosphonate hybrid materials and TiO_2_.

Scanning electron microscopy (SEM) images of the TiO_2_ sample and of the TiO_2_–octylphosphonate hybrid materials are displayed in Figure 5. The morphology of the samples did not significantly depend on the P/Ti ratio: all samples appeared to be formed of densely aggregated, roughly spherical, nanoparticles. For P/Ti ratios of 0.1 and 0.2, the particles were smaller and formed denser aggregates than for lower P/Ti ratios.

**Figure 6:**
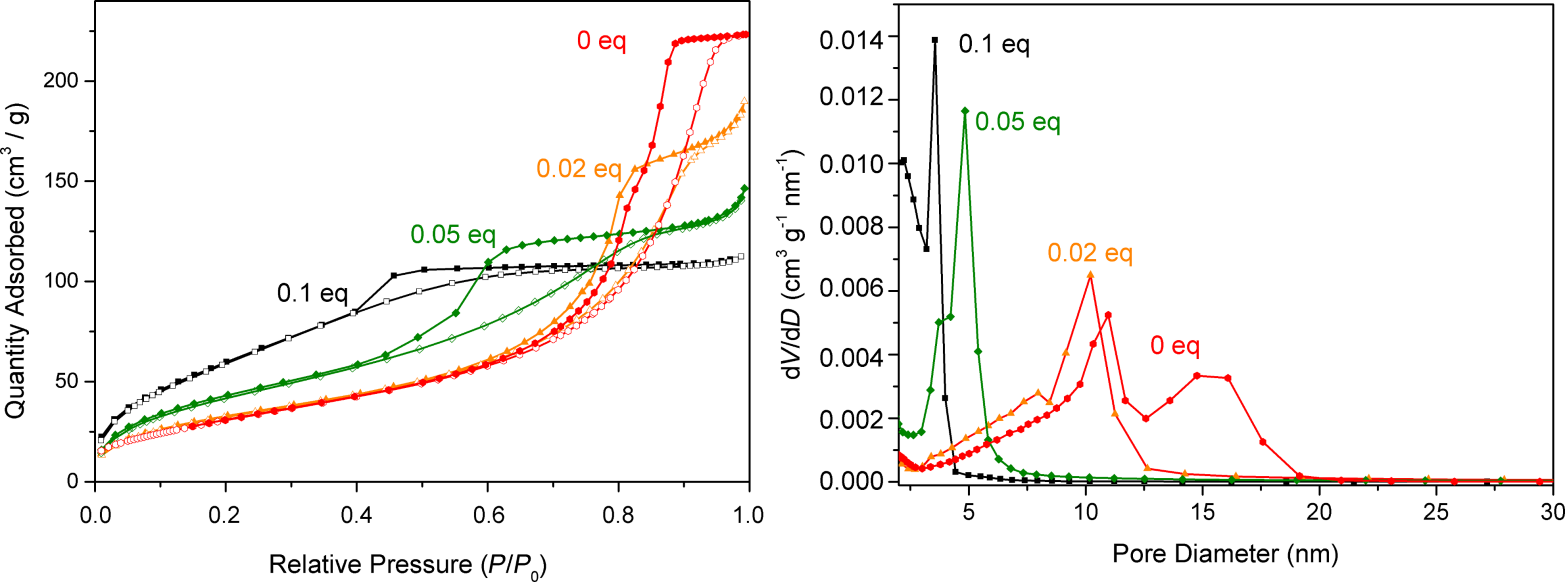
N_2_ physisorption isotherms at 77 K (left) and Barrett–Joyner–Halenda (BJH) mesopore size distribution (desorption branch, right) of TiO_2_–octylphosphonate hybrid materials and TiO_2_. Open and filled symbols in the isotherms refer to adsorption and desorption, respectively.

The nitrogen adsorption–desorption isotherms of the different samples are displayed in Figure 6, except for the TiP_0.2_ sample which was nonporous with a negligible specific surface area. All other samples showed significant porosity. Their specific surface area increased with the P/Ti ratio, from 120 m^2^ g^−1^ for TiP_0.02_ to 240 m^2^ g^−1^ for TiP_0.1_, while their pore volume decreased, from 0.29 to 0.17 cm^3^ g^−1^ (Table 1). Interestingly, the Brunauer–Emmett–Teller (BET) C constant, which is related to the adsorption enthalpy, decreased with the P/Ti ratio (42 for TiP_0.02_, 36 for TiP_0.05_, 28 for TiP_0.1_), as previously reported for nanoparticles grafted by octylphosphonic acid [37]. According to the recent IUPAC classification, the isotherms of TiO_2_ and of the hybrid samples are mainly of type IVa, characteristic of mesoporous adsorbents, with an H2 hysteresis loop indicating complex pore structures [38]. The TiP_0.02_ and TiP_0.05_ isotherms also showed Type II features (lack of plateau at high relative pressure) suggesting the presence of some macropores. As SEM images do not show the presence of macropores in TiP_0.05_ and TiP_0.02_ samples, these macropores likely correspond to pores between relatively small aggregates resulting from the grinding of the samples. The pore size distribution results confirmed the presence of mesopores in all samples (except the nonporous TiP_0.2_ sample). The sharp peak found in the distribution for TiP_0.1_ at ≈4 nm is a well-known artefact related to the instability of the meniscus at relative pressures lower than 0.42; it simply indicates the presence of small pores of diameter <4 nm. The average mesopore diameter decreased when the P/Ti ratio increased, from 8.0 nm for TiP_0.02_ to 3.1 nm for TiP_0.1_ (Table 1).

## Discussion and Conclusion

**Scheme 1:**
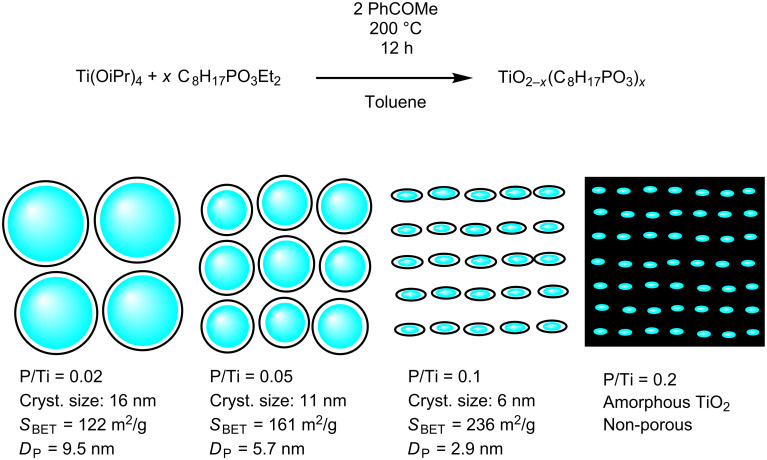
Ideal reaction scheme and hypothetical structures of the obtained hybrid materials.

The reaction at 200 °C of diethyl octylphosphonate and Ti(OiPr)_4_ in the presence of acetophenone provides a simple and original method to prepare TiO_2_–octylphosphonate hybrid materials in one step (Scheme 1).

The different characterization methods allow us to better understand the structure of these hybrid materials. The presence of even a relatively small amount of phosphonate units strongly influences the size and crystallinity of the TiO_2_ domains.

For P/Ti ratios up to 0.1, the hybrid materials can be described as rounded anatase nanoparticles grafted by octylphosphonate groups via Ti–O–P bonds. This is a major advantage of the present nonhydrolytic sol-gel method: previous attempts to prepare such TiO_2_–phosphonate hybrids by hydrolytic sol–gel routes led to amorphous TiO_2_ domains [15,39].

The low values found for the BET C constant (from 42 for TiP_0.02_ to 28 for TiP_0.1_) confirm that the surface of the anatase nanoparticles is capped by apolar octyl groups. C values of 47 and 34 have been reported for oxide nanoparticles post-modified by octylphosphonic acid with grafting densities of 1.4 and 4.1 P/nm^2^, respectively [37]. In our hybrid materials, the anatase particle size decreases with the P/Ti ratio, leading to an increase of the specific surface area of the crystallites. The density of grafting, estimated from the composition and from the diameter of the crystallites (assuming a spherical shape and a density of 3.8), increases with the P/Ti ratio from 1.5 P/nm^2^ for TiP_0.02_ to 2.9 P/nm^2^ for TiP_0.1_. These values suggest the formation of monolayers with low to moderate density (grafting densities of up to 4 to 5 P/nm^2^ have been reported for well-ordered self-assembled monolayers). The mesoporosity of the hybrid materials with P/Ti ratios up to 0.1 stems from the aggregation of the grafted nanoparticles (interparticle porosity). The smaller the size of the particles, the higher the specific surface area and the lower the pore volume (Scheme 1).

For a P/Ti ratio of 0.2, the TiO_2_ domains are completely amorphous, probably because they involve very few Ti atoms. In this case the volume fraction of the octyl groups exceeds 50%. The diameter of the TiO_2_ domains and the length of octyl chains are in the same order of magnitude, and the lack of porosity of this sample likely results from the interdigitation or mixing of the alkyl chains.

These mesoporous metal oxide–phosphonate materials can be seen as a low-cost alternative to periodic mesoporous organosilicas (PMOs) and metal–organic frameworks (MOFs) for applications in the field of heterogeneous catalysis or selective adsorption. Their high hydrolytic stability over a wide range of pH [15] and the possibility to functionalize them with a variety of functional groups makes them particularly promising for applications in aqueous phase catalysis (e.g., for biomass conversion) and in aqueous wastewater treatment.

## Experimental

Titanium(IV) isopropoxide (Ti(OiPr)_4_, 97 %), and acetophenone (99%) were obtained from Sigma-Aldrich. Diethyl 1-octylphosphonate (C_8_H_17_PO_3_Et_2_, 98%) was purchased from Sikémia. Toluene was dried over a Pure-Solve MD5 solvent purification system (H_2_O <10 ppm, controlled with a Karl Fischer coulometer). All other chemicals were used without further purification. All manipulations were carried out in a glove box under argon atmosphere (<5 ppm of water and O_2_).

### Synthesis of TiO_2_–octylphosphonate hybrids

In a typical preparation, Ti(OiPr)_4_ (1.72 g, 6.00 mmol), acetophenone (1.44 g, 12.00 mmol), C_8_H_17_PO_3_Et_2_ (0 mmol, 0.24 mmol, 0.60 mmol, 1.20 mmol, or 2.40 mmol) and toluene (8.0 mL) were mixed in a stainless steel digestion vessel with a PTFE lining (23 mL). The sealed autoclave was heated in an oven at 200 °C for 12 h under autogenous pressure. After reaction, the resulting monoliths were thoroughly washed with acetone (5 times, 30 mL). Then, they were dried under reduced pressure (5.10^−2^ mbar) at room temperature and ground into a fine powder.

### Characterization

FTIR spectra were collected in ATR mode on a Spectrum II spectrometer (Perkin-Elmer). The powder XRD patterns were collected with a PANalytical X’Pert Pro MPD diffractometer (Cu Kα_1_ = 0.1540598 nm). The SEM images were obtained with a Hitachi S-4800 electron microscope. EDX was done on an Oxford Instruments X-Max^N^ SDD instrument. Nitrogen adsorption and desorption isotherms were measured at 77 K with a Micrometrics TriStar 3000 apparatus; the specific surface area was determined by the BET method in the 0.05–0.25 *P*/*P*_0_ range. The mesopore volume and pore size distribution were obtained by the Barrett–Joyner–Halenda (BJH) method from the desorption branch.

Solid-state ^31^P magic angle spinning (MAS) NMR experiments were performed on a Varian VNMRS 400 MHz (9.4 T) spectrometer using a 3.2 mm Varian T3 HXY MAS probe. Single pulse experiments were carried out with a spinning rate of 20 kHz, a 90° excitation pulse of 3 μs, a recycle delay of 30 s and 100 kHz spinal-64 ^1^H decoupling. 200 transients were recorded. The ^31^P chemical shift was determined using an external reference, hydroxyapatite Ca_10_(PO_4_)_6_(OH)_2_, at 2.8 ppm (with respect to H_3_PO_4_, 85 wt % in water).
